# Supplementary material for: Spin-dependent quantum interference in photoemission process from spin-orbit coupled states
Source: Nat Commun. 2017 Feb 24;8:14588. doi: 10.1038/ncomms14588 (PMC5333099; doi:10.1038/ncomms14588)
Supplement: Supplementary Information — Supplementary Figures 1-2, Supplementary Notes 1-2 and Supplementary References [file ncomms14588-s1.pdf]

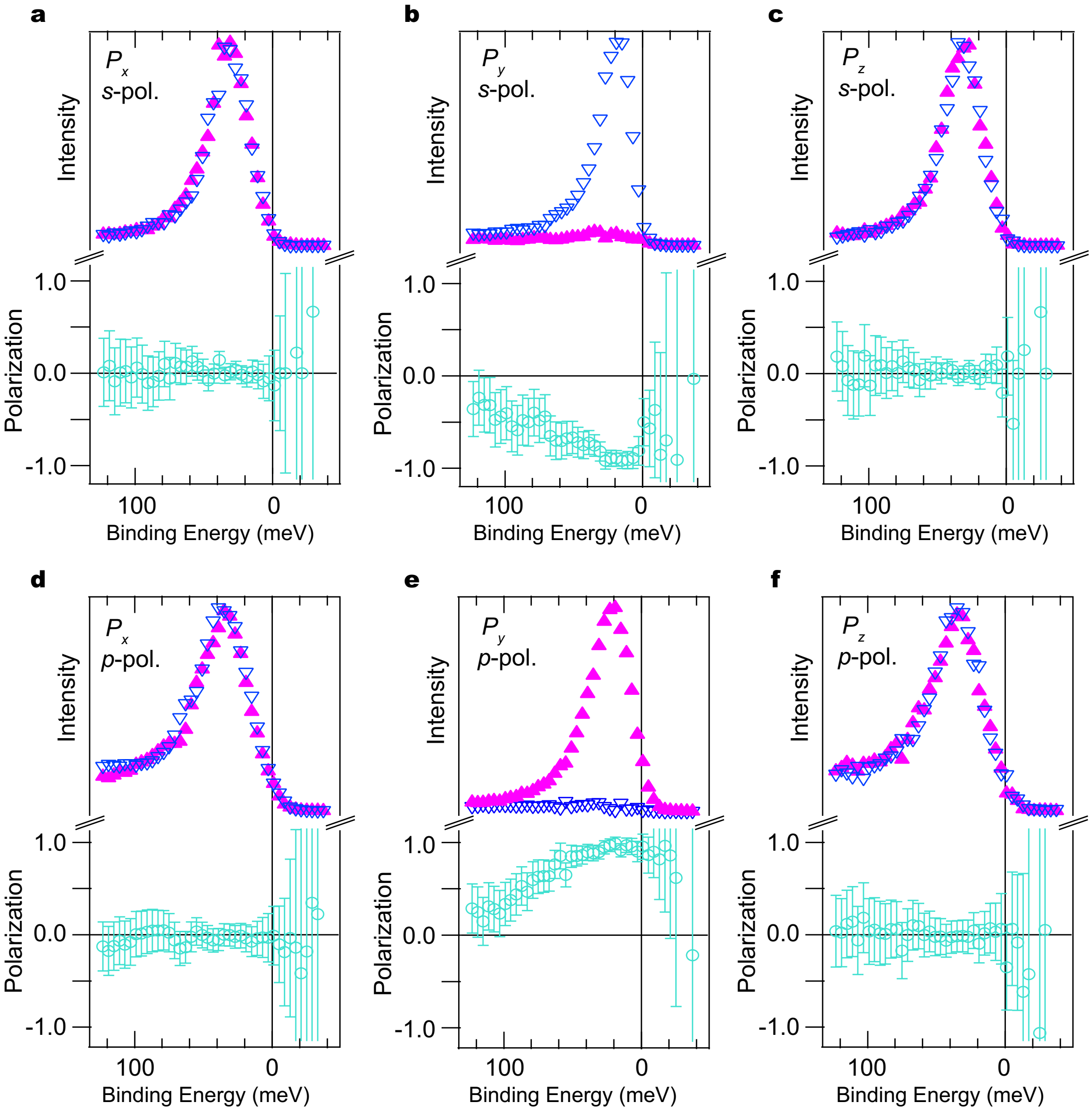

Supplementary Figure 1: **Three-dimensional SARPES of the spin-polarized surface state on Bi(111).** The spin-resolved spectra and the spin polarizations were measured with the s-polarized light (**a–c**) and the p-polarized light (**d–f**) at k_4 in Fig. 1(b) of the main text. The experimental geometry and the definition of the spin polarization axes are represented in Fig. 1(a) of the main text. The x, y and z components of the spin-resolved spectra and the spin polarization are represented by triangles and circles, respectively. The error bars represent the standard deviation of the measurements.

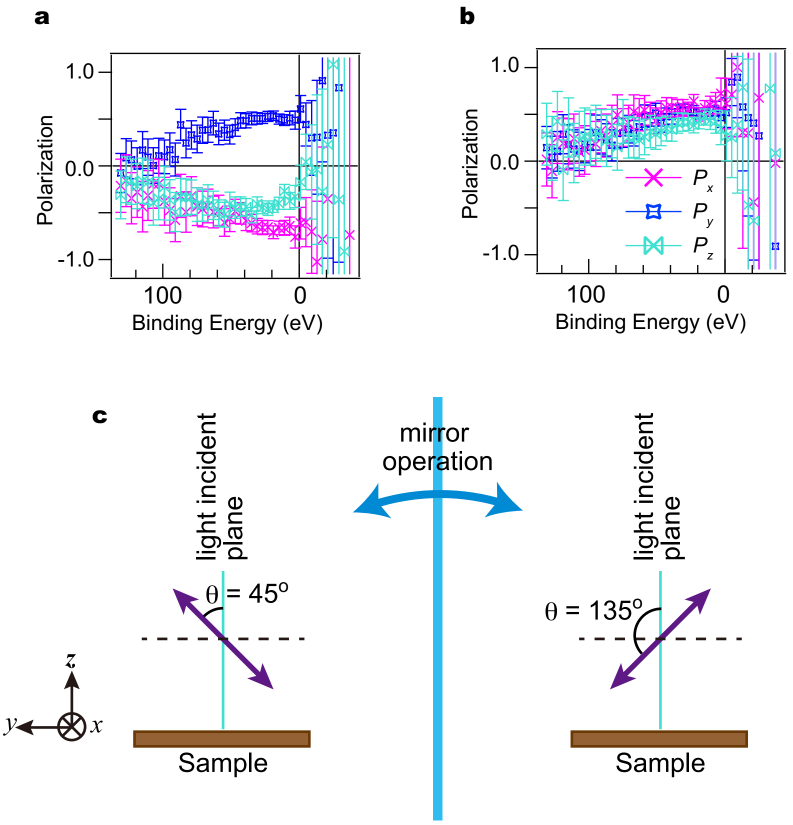

Supplementary Figure 2: **Spin polarization of photoelectron induced by breaking the mirror symmetry.** Three-dimensional spin polarization measurements with $\theta = 45^\circ$ (**a**) and $\theta = 135^\circ$ (**b**) at k_4 shown in Fig. 1(b) of the main text. The error bars represent the standard deviation of the measurements. (**c**) Schematic drawings of the experimental geometries are shown.

Supplementary Note 1. Three-dimensional spin- and angle-resolved photoelectron spectroscopy

We show typical data of three-dimensional spin- and angle-resolved photoelectron spectroscopy (SARPES) for the spin-polarized surface state on Bi(111). Supplementary Figure 1 displays the x, y and z components of spin-resolved spectra and spin polarizations at k_4 on a $\bar{\Gamma}\bar{\mathrm{M}}$ mirror plane. The light incident plane was along the $\bar{\Gamma}\bar{\mathrm{M}}$ mirror plane and the spectra were measured with s- and p-polarized lights. Thus, the experimental geometry was arranged to be symmetric. In this experimental condition, only the y component of the spin polarization is allowed as demonstrated in Supplementary Figure 1: The 100% reversal spin polarization was observed in the y direction upon switching from s- and p-polarized light while no spin polarization in the x and z directions. These results are completely consistent with a framework of the mirror symmetry and thus indicate the high accuracy of the present laser-SARPES measurements.

The absolute values of P_y at k_1 and k_4 are almost 100 %, as demonstrated in Fig. 2a,d,e,h of the main text. This is consistent with the result of the first-principles calculation shown in Fig. 3 of the main text: the $|\psi_{even}\rangle$ and $|\psi_{odd}\rangle$ states are also fully-spin-polarized at k_1 and k_4. Thus, the effects arising from the experimental geometry and the final states are apparently negligible. On the other hand, at k_2 and k_3, the absolute values of P_y at the Fermi level observed by laser-SARPES are largely reduced to the values between 20 and 80 % and not symmetric with respect to the $\bar{\Gamma}$ point. The modification of the spin-polarization can arise from the final state effect or the influence of the bulk states. In fact, the first-principles calculation in Fig. 3 of the main text also suggests the reduction of the spin polarization in the vicinity of the $\bar{\Gamma}$ point due to the existence of the bulk states.

On the other hand, the x, y and z components of the spin polarization are allowed to appear when the mirror symmetry is broken. The mirror symmetry can break by rotating the electric-field vector of the incident light with preserving the other experimental geometry. Supplementary Figure 2 is typical examples of the light-polarization-induced spin polarization, where the angles (θ) between the electric-field vector and the light incident plane are set to 45° and 135°, respectively. In this demonstration, we find the spin polarization in the x and z directions in the photoexcited states. The electric-field vectors with $\theta = 45°$ and $\theta = 135°$ are connected each other by the mirror symmetry operation. Thus, the absolute values of P_x and P_z observed $\theta = 45°$ and $\theta = 135°$ are the same, whereas the signs of P_x and P_z are switched.

Supplementary Note 2. Spin polarization on a mirror plane

We describe the spin expectation values in the initial and photoexcited states on the mirror symmetry plane. We start from the eigenstates of the mirror eigenvalues introduced in the main text:

$$\Psi_{+i} = \begin{pmatrix} \psi_{\text{even},\uparrow y} \\ \psi_{\text{odd},\downarrow y} \end{pmatrix} \text{ and } \Psi_{-i} = \begin{pmatrix} \psi_{\text{odd},\uparrow y} \\ \psi_{\text{even},\downarrow y} \end{pmatrix}. \tag{1}$$

The spin expectation values of the mirror eigenstates in the initial states are solely calculated with the Pauli matrices $\boldsymbol{\sigma}_{\text{x,y,z}}$. Note, the spin-quantization axis is defined as the direction perpendicular to the mirror plane, which corresponds to the y direction in the present system. Then, the x, y and z components of the spin expectation values for each mirror eigenstates are written as follows:

$$\begin{aligned} \langle \Psi_{+i} | \sigma_y | \Psi_{+i} \rangle &= \left\langle \psi_{\text{even},\uparrow y} \middle| \psi_{\text{even},\uparrow y} \right\rangle - \left\langle \psi_{\text{odd},\downarrow y} \middle| \psi_{\text{odd},\downarrow y} \right\rangle, \\ \langle \Psi_{+i} | \sigma_x | \Psi_{+i} \rangle &= \langle \Psi_{+i} | \sigma_z | \Psi_{+i} \rangle = 0, \end{aligned} \tag{2}$$

and

$$\begin{aligned} \langle \Psi_{-i} | \sigma_y | \Psi_{-i} \rangle &= \left\langle \psi_{\text{odd},\uparrow y} \middle| \psi_{\text{odd},\uparrow y} \right\rangle - \left\langle \psi_{\text{even},\downarrow y} \middle| \psi_{\text{even},\downarrow y} \right\rangle, \\ \langle \Psi_{-i} | \sigma_x | \Psi_{-i} \rangle &= \langle \Psi_{-i} | \sigma_z | \Psi_{-i} \rangle = 0. \end{aligned} \tag{3}$$

The Supplementary Eqs. 2,3 indicate that the net spin polarization is reduced from the maximum value of 100 % in the $|\psi_{\text{even}}\rangle$ and $|\psi_{\text{odd}}\rangle$ mixed system due to the spin-orbit interaction [1].

Next, we derive the spin expectation values of the photoexcited states [2]. Hereafter, we treat the mirror eigenvalue being $+i$ in the Supplementary Eq. 1. If we rotate the electric-field vector of the linearly polarized light between the p- and s-polarizations, the spinor field of the photoelectrons is made by simultaneous optical excitation of $\left|\psi_{\text{even},\uparrow y}\right\rangle$ and $\left|\psi_{\text{odd},\downarrow y}\right\rangle$. Here, the vector potential of the photon field (**A**) is decomposed into the p-polarization (xz-plane) and s-polarization (y-axis) components as follows:

$$\mathbf{A} = |A| \cos\theta \, \mathbf{a}_{xz} + |A| \sin\theta \, \mathbf{a}_y, \tag{4}$$

where $\mathbf{a}_{xz}$ and $\mathbf{a}_y$ are unit vectors of the decomposed vector potential. Then, the dipole transition matrix elements of photoemission for the even and odd parity parts can be written as

$$m_{\mathrm{even},\uparrow y} = \langle\psi_{\mathrm{final}}|\,\mathbf{A}\cdot\mathbf{p}\,\left|\psi_{\mathrm{even},\uparrow y}\right\rangle,$$
$$m_{\mathrm{odd},\downarrow y} = \langle\psi_{\mathrm{final}}|\,\mathbf{A}\cdot\mathbf{p}\,\left|\psi_{\mathrm{odd},\downarrow y}\right\rangle, \tag{5}$$

where $\mathbf{p}$ is the momentum operator and ψ_{final} is a final-state wavefunction. Here, we assume that the final photoelectron state is spin-degenerated. Thus, the spinor field of the photoelectrons χ_{p} is described by

$$\left|\chi_{\mathrm{p}}\right\rangle = \begin{pmatrix} m_{\mathrm{even},\uparrow y}\cos\theta\,\psi_{\mathrm{final}} \\ m_{\mathrm{odd},\downarrow y}\sin\theta\,\psi_{\mathrm{final}}. \end{pmatrix}. \tag{6}$$

The spin expectation values $P_{x,y,z}$ of the photoexcited states $\left|\chi_{\mathrm{p}}\right\rangle$ are obtained with Pauli matrices $\boldsymbol{\sigma}_{\mathrm{x,y,z}}$ as follows:

$$P_x = \frac{i\left(m_{\mathrm{even},\uparrow y}m^*_{\mathrm{odd},\downarrow y} - m_{\mathrm{odd},\downarrow y}m^*_{\mathrm{even},\uparrow y}\right)}{I_{\mathrm{total}}}\cos\theta\sin\theta,$$

$$P_y = \frac{\left|m_{\mathrm{even},\uparrow y}\right|^2\cos^2\theta - \left|m_{\mathrm{odd},\downarrow y}\right|^2\sin^2\theta}{I_{\mathrm{total}}},$$

$$P_z = \frac{m_{\mathrm{even},\uparrow y}m^*_{\mathrm{odd},\downarrow y} + m_{\mathrm{odd},\downarrow y}m^*_{\mathrm{even},\uparrow y}}{I_{\mathrm{total}}}\cos\theta\sin\theta,$$

$$I_{\mathrm{total}} = \left|m_{\mathrm{even},\uparrow y}\right|^2\cos^2\theta + \left|m_{\mathrm{odd},\downarrow y}\right|^2\sin^2\theta. \tag{7}$$

Note, the $P_{x,y,z}$ obey the spin conservation law of $|\mathbf{P}| = \sqrt{{P_x}^2 + {P_y}^2 + {P_z}^2} = 1$. To simplify the Supplementary Eq. 7, we introduce the ratio of the matrix elements $\frac{m_{\mathrm{odd},\downarrow y}}{m_{\mathrm{even},\uparrow y}} = ue^{i\alpha}$. Then, the Supplementary Eq. 7 can be changed as follows:

$$P_x = \frac{2\left|m_{\mathrm{even},\uparrow y}\right|\left|m_{\mathrm{odd},\downarrow y}\right|\sin\alpha\sin\theta\cos\theta}{\left|m_{\mathrm{even},\uparrow y}\right|^2\cos^2\theta + \left|m_{\mathrm{odd},\downarrow y}\right|^2\sin^2\theta} = \frac{2u\sin\alpha\tan\theta}{1+u^2\tan^2\theta},$$

$$P_y = \frac{\left|m_{\mathrm{even},\uparrow y}\right|^2\cos^2\theta - \left|m_{\mathrm{odd},\downarrow y}\right|^2\sin^2\theta}{\left|m_{\mathrm{even},\uparrow y}\right|^2\cos^2\theta + \left|m_{\mathrm{odd},\downarrow y}\right|^2\sin^2\theta} = \frac{1-u^2\tan^2\theta}{1+u^2\tan^2\theta},$$

$$P_z = \frac{2\left|m_{\mathrm{even},\uparrow y}\right|\left|m_{\mathrm{odd},\downarrow y}\right|\cos\alpha\sin\theta\cos\theta}{\left|m_{\mathrm{even},\uparrow y}\right|^2\cos^2\theta + \left|m_{\mathrm{odd},\downarrow y}\right|^2\sin^2\theta} = \frac{2u\cos\alpha\tan\theta}{1+u^2\tan^2\theta},$$

$$\frac{I_{\mathrm{total}}(\theta)}{I_{\mathrm{total}}(\theta=0)} = \cos^2\theta + u^2\sin^2\theta. \tag{8}$$

The Supplementary Eq. 8 corresponds to the formulas shown in the main text. The maxima of P_x and P_z are given by $\sin\alpha$ and $\cos\alpha$, respectively. In addition, we find the following relationship from the Supplementary Eq. 8;

$$\frac{P_x}{P_z} = \tan\alpha. \tag{9}$$

Thus, we can readily obtain the phase difference α of the dipole matrix elements between the even and odd parity states by three-dimensional SARPES with the linearly polarized light. We emphasize that the Supplementary Eq. 8 reproduces the nature of the spin polarization of photoelectron with only two parameters.

Supplementary References

[1] Yazyev, O. V., Moore, J. E. and Louie S. G. Spin polarization and transport of surface states in the topological insulators Bi_2Se_3 and Bi_2Te_3 from first principles. *Phys. Rev. Lett.* **105**, 266806 (2010).

[2] Kuroda, K. *et al.* Coherent control over three-dimensional spin polarization for the spin-orbit coupled surface state of Bi_2Se_3. *Phys. Rev. B*, **94**, 165162 (2016).
